# Prognostic role of HPV infection in esophageal squamous cell carcinoma

**DOI:** 10.1186/s13027-018-0210-9

**Published:** 2018-11-29

**Authors:** Laura Bognár, Ivett Hegedűs, Szabolcs Bellyei, Éva Pozsgai, László Zoltán, Katalin Gombos, Örs Péter Horváth, András Vereczkei, András Papp

**Affiliations:** 1 Department of Surgery, Medical School, University of Pécs, Hungary, Ifjuság Street 13, Pécs, 7624 Hungary; 2 Department of Pathology, Medical School, University of Pécs, Hungary, Szigeti Street 12, Pécs, 7624 Hungary; 3 Department of Oncotherapy, Medical School, University of Pécs, Hungary, Édesanyák Street 17, Pécs, 7624 Hungary; 4 Department of Primary Health Care, Medical School, University of Pécs, Hungary, Rákóczi Street 2, Pécs, 7623 Hungary; 5 Department of Laboratory Medicine, Medical School, University of Pécs, Hungary, Szigeti Street 12, Pécs, 7624 Hungary

**Keywords:** Human papillomavirus, Esophageal squamous cell carcinoma, Neoadjuvant therapy, Heat shock protein, Growth hormone-releasing hormone receptor

## Abstract

**Background:**

The aims of this study were to evaluate whether HPV infection has a prognostic role in patients with esophageal squamous cell carcinoma who underwent oncological treatment and also to compare the heat shock proteins (Hsp) 90, 27 and 16.2 and growth hormone-releasing hormone receptor (GHRH-R) expression patterns of the pre-treatment tumor biopsies with the HPV status and with the oncological response.

**Methods:**

Pre-treatment tumor biopsies of 74 patients with locally advanced esophageal squamous cell carcinoma were processed retrospectively. The presence of HPV was detected by chromogenic in situ hybridization. Hsp and GHRH-R expressions were determined using immunohistochemistry. Following neoadjuvant or definitive radiochemotherapy, the patients were restaged according to the Response Evaluation Criteria in Solid Tumors. The correlation between the HPV status, response to treatment and Hsp and GHRH-R expressions were evaluated.

**Results:**

Fourteen (19%) patients were HPV-positive. These patients were more likely to respond poorly to multimodal therapy (71.4% were non-responders vs. 28.6% responders) and had shorter survival compared to HPV-negative patients (mean survival of 8 months vs. 11 months), although the difference was not significant. A significantly higher number of HPV-positive patients expressed Hsp 90 and 16.2 at high levels (93 and 79%, respectively) than at low levels (Chi-Square *p* = 0.019 and *p* = 0.031). Higher levels of Hsp expressions were associated with poorer response to therapy and worse overall survival. No correlation was found between GHRH-R expression and the HPV status, nor between GHRH-R expression and the treatment response of the examined samples.

**Conclusions:**

We found that HPV infection was associated with poor response to oncological treatment and decreased overall survival, and therefore proved to be a negative prognostic factor in patients with esophageal squamous cell carcinoma. There was a linear correlation between levels of Hsp 90 and 16.2 expression and HPV positivity.

## Background

Esophageal cancer is the eighth most common malignant tumor and the sixth leading cause of cancer mortality worldwide, with approximately 500.000 new cases diagnosed and an estimated 406.000 deaths each year [1]. Esophageal cancer has a poor prognosis with a 5-year survival rate around 15–20%, mainly due to the absence of early symptoms and therefore late stage diagnosis [2]. Despite increasing rates of esophageal adenocarcinoma in many western countries, esophageal squamous cell carcinoma (ESCC) remains the dominant histological type of esophageal cancer globally. The incidence of squamous cell carcinoma of the esophagus varies considerably from place to place, suggesting an important role of environmental factors in its etiology [3]. The main risk factors involved in the etiology of the disease are well established, including alcohol and tobacco consumption and low socioeconomic status. The hypothesis that HPV could potentially be involved in the pathogenesis of esophageal malignancies was first proposed by Syrjänen et al. in 1982 [4]. Since then, the connection between HPV infection and esophageal squamous cell carcinoma has been widely studied. Several systematic reviews and meta-analyses have been published recently that observed a close association between HPV infection and the incidence of ESCC [5–8]. However, the presumed underlying oncogenic mechanisms of HPV-induced esophageal squamous cell carcinoma are poorly understood, and until now, the International Agency on Research on Cancer has not made a definite statement on the potential etiologic relationship between HPV and ESCC.

The impact of HPV infection on response to the oncological treatment and survival is not fully elucidated yet. In locally advanced esophageal cancer neoadjuvant chemoradiotherapy (CRT) can downsize the primary lesion, decrease the potential for metastasis, increase the resectability rate and consequently improve long-term survival [9, 10]. It is well known, that patients with locally advanced esophageal cancer respond differently to neoadjuvant therapy, due to unexplained factors. In a previous study we found that with neoadjuvant CRT complete pathological response was achieved in 17% of patients and, partial response in approximately the half, while in 16% of the cases stable disease and in 15% progression was observed [11]. Significant improvement in long-term survival can only be expected in patients who have complete pathological response, emphasizing the need for finding prognostic markers that can distinguish between the responder and non-responder group, and consequently save non-responder patients from unnecessary overtreatment with cytostatics. So far no clinically relevant markers have been found that could predict the response to preoperative therapy. Expression levels of stress-inducible heat shock proteins (Hsp) are well-known to be altered during malignant transformation, either increasing or decreasing [12], and studies have also shown that the expression of heat shock proteins is closely related to the prognosis of carcinomas [13, 14]. However, limited and inconsistent reports exist on the relationship between Hsp expression and response to neoadjuvant chemoradiotherapy in ESCC patients. Furthermore, to our knowledge, there have been no studies investigating the association between HPV infection and heat shock protein expression patterns in ESCC patients. Similarly, growth hormone-releasing hormone receptors (GHRH-R) have been found in a variety of tumoral tissues and cell lines and their expression levels proved to be an independent predictor of patient prognosis [15].

The present study aimed to evaluate the effect of tumor HPV status on the prognosis and response to CRT in patients with ESCC. It was also our goal to investigate the correlation between the expressions of Hsp-s (90, 27,16.2), GHRH-R and response to therapy and overall survival.

## Patients and methods

### Patients

A retrospective histological examination of pre-treatment tumor tissue samples from patients with locally advanced esophageal squamous cell carcinoma was carried out. All patients received chemoradiotherapy at the Department of Oncotherapy, Clinical Center, University of Pécs, Hungary, between 2006 and 2016. Following oncological treatment, patients either underwent surgery or continued chemoradiotherapy. Inclusion criteria required that all examinations and treatments of the patients had to be carried out in the Clinical Center of the University of Pécs. 80 patients were originally enrolled in the study, 6 patients were subsequently excluded for different reasons, leaving us with 74 valid patients. Regarding sex ratio a high male dominance was observed (58 males,16 females). 12 patients had upper third, 41 patients had middle third and 21 patients had lower third esophageal tumor. All patients had squamous cell cancer, with stages cT3–4, cN0–2, cM0–1. The staging procedure included endoscopy, endoscopic ultrasound, chest X-ray, computed tomography (CT) and bronchoscopy with brush cytology. As oncological treatment, patients received CT planned external-beam radiotherapy (180 cGy daily for 5 days weekly up to 39.6–45 Gy) and concomitant chemotherapy during the first week of irradiation: cisplatin (60–100 mg/m^2^) on day 1, 5-fluorouracil (750–1000 mg/m^2^/day) and Ca-folinat (20 mg/m^2^/day) infusions on days 1–5. After a six-week-long treatment-free period, restaging was carried out according to the Response Evaluation Criteria in Solid Tumors (RECIST) [16]. In order to simplify the evaluation of the results, patients were divided into two groups: *responders* including patients who showed complete or partial response and *non-responders* including patients where either stable disease or disease progression were observed.

### HPV detection

Sections from the pre-treatment tumor tissue samples were fixed in formalin and embedded in paraffin. The presence of HPV was detected by chromogenic in situ hybridization (CISH) using ZytoFast PLUS Implementation Kits. Briefly, this system detects HPV types 6, 11, 16, 18, 31, 33, 35, 45, 51 and 82 using Digoxigenin-labeled probes which are detected using primary antibodies. These antibodies are then detected by polymerized enzyme-conjugated secondary antibodies. The enzymatic reaction of chromogenic substrates leads to the formation of strong color precipitates that can be visualized by light microscopy.

Tissue samples that were positive for HPV by CISH were subsequently genotyped using the Linear Array HPV Genotyping Test (Cat. No: 04391853190, Roche Diagnostics, Mannheim, Germany) according to the accredited molecular biological routine method in the Molecular Genetic Laboratory of the Department of Laboratory Medicine, University of Pécs (accreditation number: NAH molecular biology diagnostics L7–1 MLMB01, Roche Linear Array HPV Genotyping Test). This test can simultaneously detect up to 37 different HPV genotypes in one sample.

### Immunohistochemical staining for Hsp 90, 27 and 16.2 and GHRH-R

Immunohistochemical reactions were carried out by LEICA BOND automated staining machines, using polyclonal rabbit antibodies directed against the human Hsp 90, 27, 16.2 and GHRH-R. The formalin-fixed, paraffin-embedded tumor biopsies were sliced into 4 μm thick sections and dried in a 56 C thermostat for 2 h. The tissue sections were deparaffinized with Bond Dewax Solution, for 8 min. Antigen retrieval was carried out with a Bond Epitope Retrieval Solution for 20 min, at 97 C and pH 6.00. The conditions were identical for each of the 4 antibodies. The specimens were then incubated with the primary antibodies at 42 C, for 15 min, according to the following dilutions: Hsp 90–1:100; Hsp 27–1:4000; Hsp 16.2–1:1000; GHRH-R: 1:50. The sections were then incubated with the secondary antibodies at 42 C, for 10 min, without dilution and they were counterstained with hematoxylin and eosin. The immunoreactions were visualized by Bond Polymer Refine Detection. The tissue sections were dehydrated through an ascending series of alcohol, then covered in xylene and Pertox.

The immunostaining was interpreted by our pathologist blindly, without knowledge of the treatment response rate of the patients. The presence of cytoplasmic staining with or without nuclear staining was required to assign the positivity for heat shock proteins and GHRH-R. Staining intensity was sorted into three categories: 1. samples that showed homogenous strong expression of the antigens 2. samples that showed heterogenous staining pattern with typically weaker staining intensity 3. samples that showed no staining at all. For easier evaluation these categories were then simplified into two classes: *high-intensity* (1) and *low-intensity* (2 + 3) samples.

### Statistical analysis

Finally, the correlations between the HPV status and response, and between the HPV status and the different biomarkers were established with the Chi-Square test. Survival rates of the HPV positive and negative groups were estimated using the Kaplan-Meier method, and compared with the log-rank test. A *p-value* < 0.05 was considered significant for the comparison. For the statistical analysis the IBM SPSS Statistics v23 software package was used.

## Results

### Clinical and patient data

Of the 74 patients participating in the study, 22 patients (30%) received neoadjuvant CRT and 52 patients (70%) received definitive CRT due to their general condition and/or advanced stage of the disease. 38 patients (51%) responded well to therapy. Ultimately, 14 out of the 22 patients, who had neoadjuvant CRT, underwent surgical resection. Reasons for not having surgery included: not responding well to CRT (5 patients), refusing to consent to surgery (2 patient) and death (1 patient). Fourteen (19%) of the 74 ESCC patients were found to be HPV positive. Regarding the distribution of the sexes and locations of the tumors, in the HPV positive group we found a male:female ratio of 8:6, where 4 patients had upper third, 6 patients had middle third and 4 patients had lower third esophageal tumor, while in the HPV negative group the male:female ratio was 5:1, and among them 8 patients had upper third, 35 had middle third and 17 had lower esophageal tumor. Baseline characteristics of HPV-positive and HPV-negative patients are shown in Table 1.

**Table 1:** Characteristics of HPV negative versus HPV positive patients

Variable		HPV negative	HPV positive
		(*n = 60*) no. (%)	(*n = 14*) no. (%)
Age at diagnosis	≤ 60	29 (48.3%)	4 (28.6%)
	> 60	31 (51.7%)	10 (71.4%)
Gender	Female	10 (16.7%)	6 (42.9%)
	Male	50 (83.3%)	8 (57.1%)
Tumor location	Upper third	8 (13.3%)	4 (28.6%)
	Middle third	35 (58.3%)	6 (42.8%)
	Lower third	17 (28.4%)	4 (28.6%)
Clinical T stage	cT3	26 (43.3%)	8 (57.1%)
	cT4	34 (56.7%)	6 (42.9%)
Clinical N stage	cN0	6 (10.0%)	4 (28.6%)
	cN1–2	54 (90.0%)	10 (71.4%)
Clinical M stage	cM0	49 (81.7%)	14 (100.0%)
	cM1	11 (18.3%)	0 (0.0%)

We also aimed to detect HPV DNA sequence in our positive cases to examine the distribution of HPV genotypes. Unfortunately, repeated linear array tests failed to give results for proper laboratory evaluation. This might be due to overfixation of the small tissue samples in formaldehyde, which resulted in the damage and degradation of the DNA.

### The effects of HPV status on response to therapy and prognosis

**Fig. 1:**
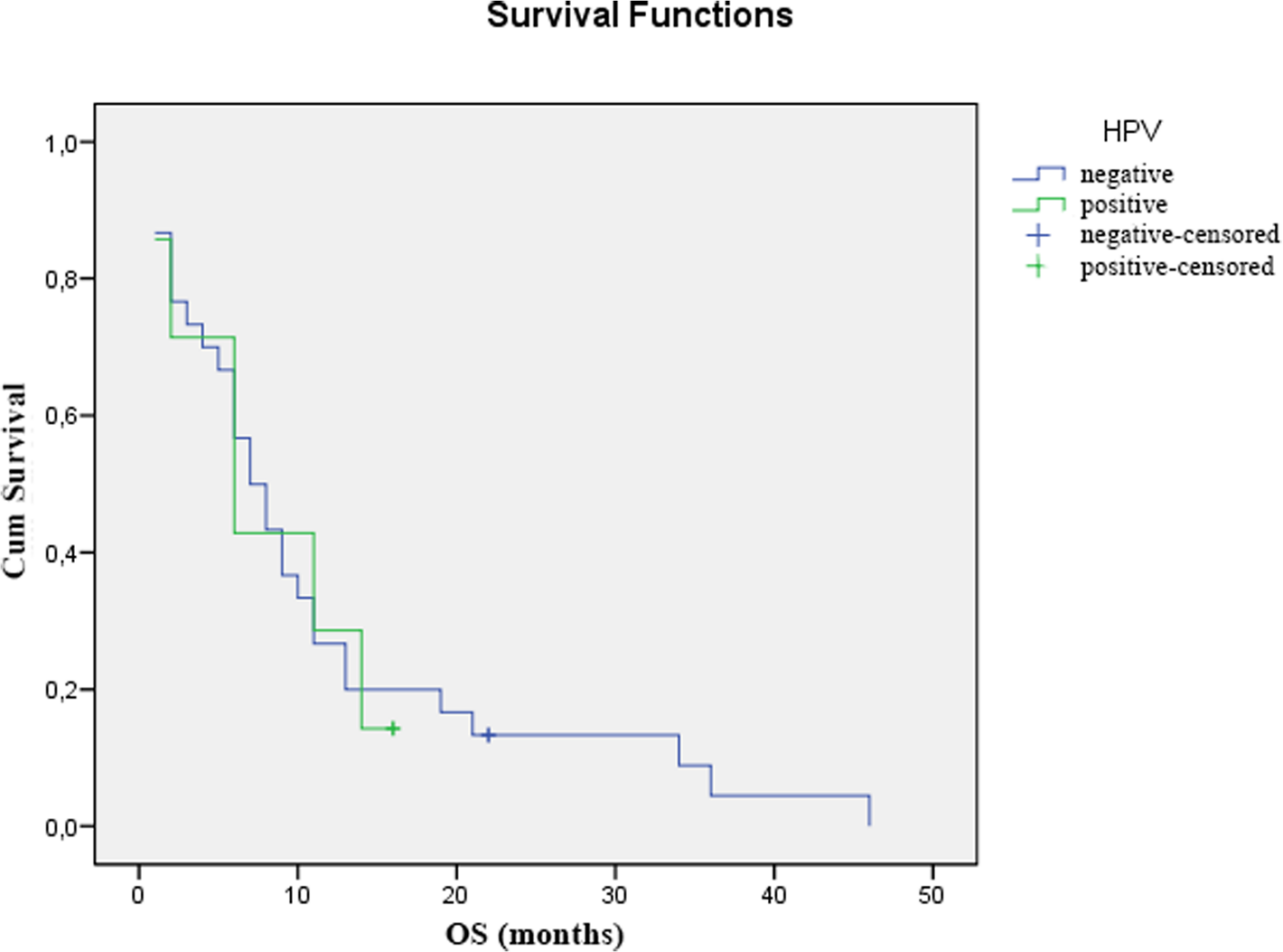
The effect of HPV status on overall survival demonstrated with Kaplan-Meier curve

Comparing the HPV status and the clinical response to CRT, we found that HPV positivity was associated with a higher rate of non-responder patients (71.4% non-responders vs. 28.6% responders), however, this difference was not significant (Chi-Square *p* = 0.058) (Table 2). Similarly, the overall survival of HPV-positive patients was shorter compared to HPV-negative patients (mean survival of 8 months vs. 11 months and median survival of 6 months vs. 7 months), but this difference was also not significant (log-rank *p* = 0.898) (Fig. 1).

**Table 2:** The effect of HPV status on response to CRT

		Clinical Downstaging (*n* = 74)		*p value*
		Responder	Non-responder	
HPV	negative	34 (56.7%)	26 (43.3%)	*p = 0.058*
	positive	4 (28.6%)	10 (71.4%)

### The relationship between HPV status and expressions of Hsp 16.2, 27, 90 and GHRH-R

Significantly more HPV positive tumors expressed Hsp 90 and 16.2 at high intensities than at low intensities (Chi-Square *p* = 0.019 and *p* = 0.031). On the other hand, there was a near-equal distribution of low and high intensity Hsp staining in HPV negative tumors. No significant correlation could be observed between the Hsp 27 and GHRH-R expression patterns and HPV positivity (Table 3).

**Table 3:** The relationship between HPV status and expressions of Hsp 16.2, 27, 90 and GHRH-R

Molecular Marker		HPV status (*n* = 74)		*p value*
		negative	positive	
Hsp 16.2	low intensity	32 (53.3%)	3 (21.4%)	***p = 0.031***
	high intensity	28 (46.7%)	11 (78.6%)	
Hsp 27	low intensity	30 (50.0%)	6 (42.9%)	*p = 0.630*
	high intensity	30 (50.0%)	8 (57.1%)	
Hsp 90	low intensity	24 (40.0%)	1 (7.1%)	*p = 0.019*
	high intensity	36 (60.0%)	13 (92.9%)	
GHRH-R	low intensity	42 (70.0%)	8 (57.1%)	*p = 0.355*
	high intensity	18 (30.0%)	6 (42.9%)	

### The effects of Hsp expression on response to therapy

**Fig. 2:**
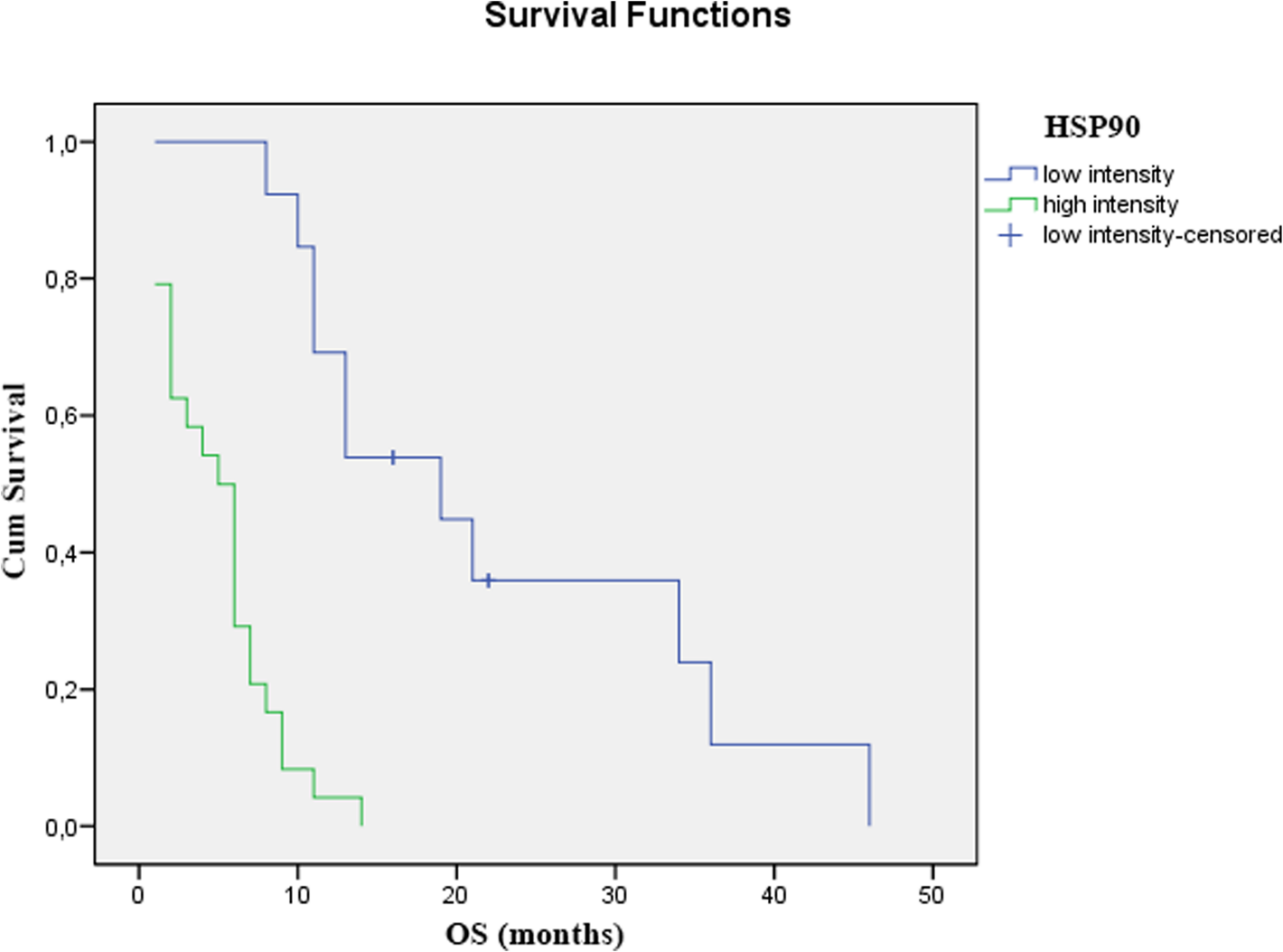
The effect of Hsp 90 expression on overall survival is demonstrated using a Kaplan-Meier curve and the level of significance is determined using the log-rank test. Probability (*p*) values < 0.05 are considered statistically significant. *p* < 0.001

**Fig. 3:**
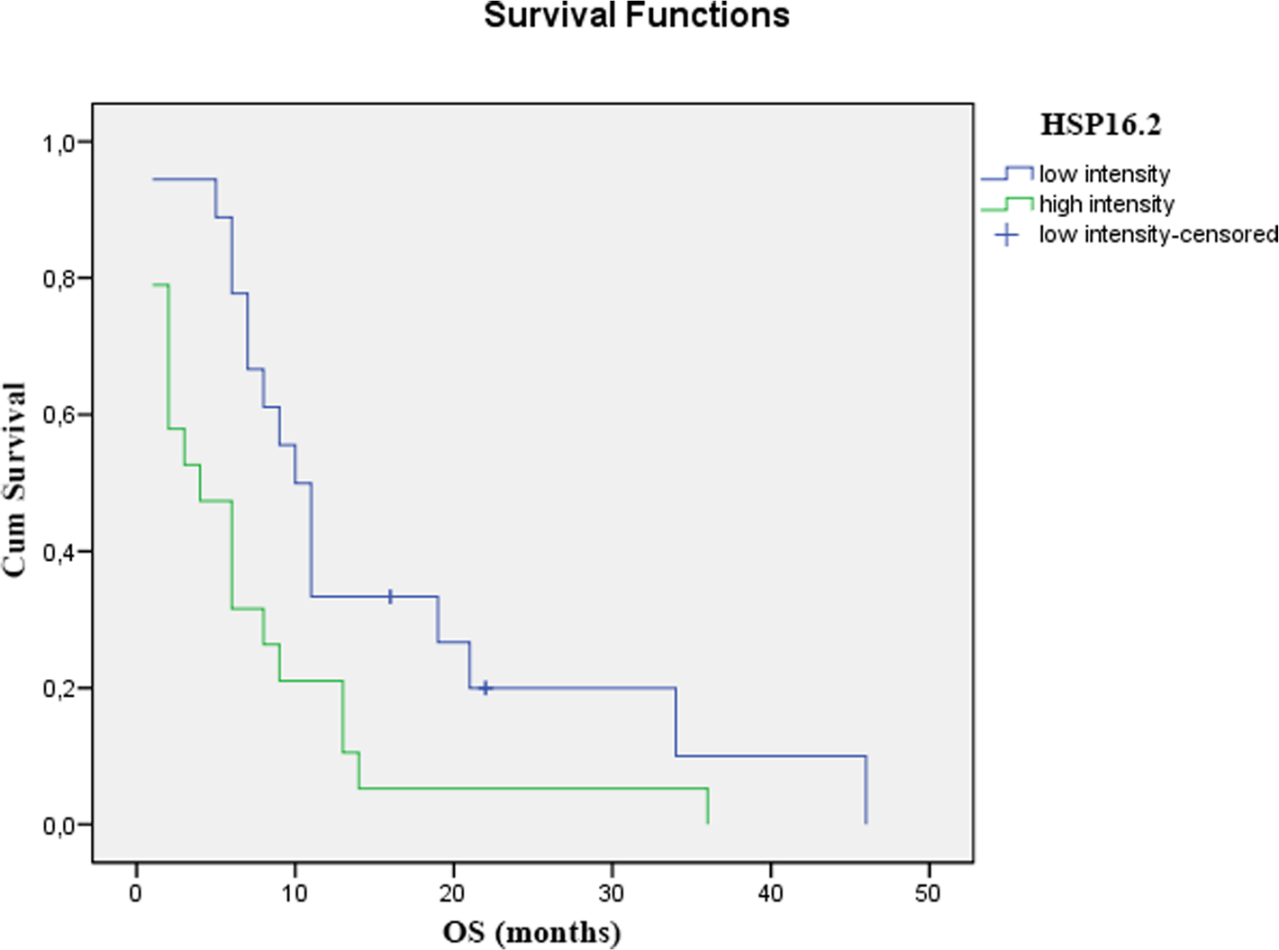
The effect of 16.2 expression on overall survival is demonstrated using a Kaplan-Meier curves and the level of significance is determined using the log-rank test. Probability (*p*) values < 0.05 are considered statistically significant. *p* < 0.001

Among non-responders, there were significantly more tumors, which expressed Hsp 90 and 16.2 at high levels (Chi-Square *p* < 0.001 and *p* < 0.01). This tendency was also apparent in the expression levels of Hsp 27, but the difference was not significant (Table 4). We also found that patients with tumors that expressed Hsp-s at high levels had a significantly shorter overall survival, than patients with tumors that stained low for Hsp-s. (Figs. 2 and 3).

**Table 4:** The effects of Hsp expression on response to CRT

Molecular Marker		Clinical Downstaging (*n* = 74)		*p value*
		Responder	Non-Responder	
Hsp 16.2	low intensity	29 (82.9%)	6 (17.1%)	*p < 0.01*
	high intensity	9 (23.1%)	30 (76.9%)	
Hsp 27	low intensity	22 (61.1%)	14 (38.9%)	*p = 0.102*
	high intensity	16 (42.1%)	22 (57.9%)	
Hsp 90	low intensity	21 (84.0%)	4 (16.0%)	*p < 0.001*
	high intensity	17 (34.7%)	32 (65.3%)

## Discussion

In recent years, a large number of studies have investigated human papillomavirus infection in esophageal squamous cell carcinoma, with largely inconclusive results [5–8]. The detection rates of HPV-ESCC show high variability worldwide. There are large geographic differences in the overall incidence of ESCC, with high-incidence countries within the ‘Asian esophageal cancer belt’ reporting up to one-hundred-fold higher rates of ESCC compared to low-incidence countries, such as the United States, Europe or Australia [17]. In high-ESCC-incidence countries, the HPV detection rate in tumor tissues is also significantly higher compared to low-ESCC-incidence countries (32.8–63.6% in China vs. 8.7–16.6% in North America) [18]. In our study, among the Hungarian population, the rate of HPV positivity in ESCC patients corresponded to that of the low risk countries. Namely, 14 (19%) out of the 74 patients were confirmed to be HPV positive by CISH. The prognostic value of HPV status has previously been investigated in patients with ESCC [19–21]. The first meta-analysis investigating overall survival in HPV-related esophageal cancer was published in 2016, and showed no significant association between HPV infection and survival [22]. The controversial results of the included studies are in contrast to oropharyngeal lesions, where HPV-positivity has been consistently shown to be a strong positive prognostic factor in patient outcomes [23, 24]. A potential correlation between HPV infection and response to neoadjuvant treatment in patients with ESSC has also been the subject of ongoing debate. Several studies demonstrated that HPV-positive cervical cancer patients had significantly better clinical response to oncological treatment and survived significantly longer than HPV negative patients [25, 26]. Therefore, HPV can be considered an independent prognostic parameter for radiosensitivity and survival in patients with cervical cancer. HPV infection in esophageal cancer as a possible predictive factor after neoadjuvant therapy has been studied before [27, 28]. Bognár et al. and Wang et al. found a correlation between HPV infection and response to CRT. They both reported that HPV positive patients responded better to CRT and had a significantly more favorable survival compared to the HPV negative group; however, due to the relatively low number of patients involved, far-reaching conclusions could not be drawn. In our study, we found an opposite result, namely that the HPV-positive group responded worse to CRT and had worse overall survival than the HPV-negative group. Therefore, in our study, HPV positivity was a negative prognostic factor in relation to multimodal therapy and to overall survival, though the differences were not significant.

In our study we also examined the anti-apoptotic Hsp 90, 27 and 16.2 expression patterns in the pre-treatment tumor biopsies. Heat shock proteins are induced in response to a wide variety of physiological and environmental insults, thus allowing cells to survive lethal conditions based on their cytoprotective functions. Associated with key apoptotic factors, they are powerful anti-apoptotic proteins, having the capacity to block cell death process at different levels. Hsp overexpression signals a poor prognosis in terms of survival and response to therapy in specific cancer types [29, 30]. In a previous study, we examined the Hsp 90 and 16.2 expression of esophageal tumor specimens prior to CRT, in search of possible predictive biomarkers of response to multimodal therapy [31]. We found that the tumor samples from the patients with no clinical response contained approximately double the level of Hsp 90 and 16.2, significantly higher than the responding tumors. In the present study, we examined whether HPV infection, as an environmental insult, influences the Hsp expression pattern of ESCC patients. We found elevated Hsp 90 and 16.2 expression levels in the HPV-positive tumor samples compared to the HPV-negative ones. As expected, increased levels of Hsp 90 and 16.2 expression, were associated with significantly poorer response to CRT and worse overall survival. It is unclear why HPV positivity in ESCC patients proves to be a negative prognostic marker in certain regions of the world, while in others it is a positive prognostic marker. In head and neck tumors, the development of cancer is attributed to different oncogene mechanisms in patients with HPV positive tumors and in those who don’t carry the virus but have a dominant history of alcohol and tobacco consumption [32]. The biology of HPV-positive oropharyngeal cancer is characterized by p53 degradation, retinoblastoma Rb pathway inactivation, and p16 upregulation, while, by contrast, tobacco-related oropharyngeal cancer is characterized by TP53 mutation and downregulation of CDKN2A (encoding p16) [33]. It is also well known that HPV-positive oropharyngeal cancers seem to be more responsive to chemotherapy and radiation than HPV-negative tumors. In line with these findings, we have set up a hypothesis, which could explain why HPV-positive esophageal squamous cell cancers respond differently to multimodal therapy. In regions where the HPV detection rate in esophageal tumors is high, a positive correlation can be observed between HPV positivity and response to treatment. We presume that in these cases the viral infection plays a role in the cancerogenesis itself, while in the low-risk regions, the development of cancer is attributed to other factors, such as poor socioeconomic environment, excessive alcohol and tobacco consumption, and superinfection of the esophagus by HPV. The evaluation of p16 expression, a surrogate biomarker for HPV infection, is also of importance regarding prognosis of ESCC. Expression of p16 in ESCC means an active HPV infection in tumor cells and has been shown to correlate with higher rate of pathologic complete remission in patients undergoing neoadjuvant chemotherapy [34], compared with p16 negative individuals, who carry HPV DNA. We presume that this may be attributed to the fact that in p16 positive individuals the virus itself induced the cancerogenesis, while in p16 negative cases that were HPV DNA positive, HPV means only a superinfection of the tumorous cells. This superinfection, as an environmental insult, could lead to an increased expression of heat shock proteins, and as a consequence these tumors respond worse to anticancer treatments. This hypothesis brings up an unanswered issue of the current understanding of the epidemiology and biology of HPV-associated esophageal squamous cell carcinoma. Evaluation of p16 expression besides HPV DNA status would be thus imperative in pre-treatment ESCC samples to differentiate between an active and a passenger HPV infection and consequent, potentially different prognosis. Unfortunately, in our study analysis of p16 expression could not be undertaken due to the degradation of the tissue samples, leading to major limitation of our study. As such, to confirm this hypothesis further, prospective studies are needed.

Today, the Advisory Committee on Immunization Practices (ACIP) recommends routine HPV vaccination for females and males at age 11 or 12 years, to prevent infection with HPV types that are associated with certain cancers, including cervical, vaginal, vulvar, anal, throat and penile cancers [35]. The recommendation doesn’t comprise the prevention of HPV-associated esophageal cancers, however, growing literature demonstrates that the virus is involved in the development of esophageal squamous cell carcinoma or may worsen the prognosis. In our opinion, extension of the indications of prophylactic immunization is imperative.

Growth hormone-releasing hormone (GHRH) is a peptide hormone secreted by the hypothalamus, but it is also present in various tissues and tumors, stimulating the secretion of growth hormone (GH) after binding to pituitary-type GHRH receptors (GHRH-R) on the anterior pituitary. GH stimulates the production of the insulin-like growth factor I (IGF-I), which plays a major role in malignant transformation, metastasis and tumorigenesis in various cancers [36]. The presence of GHRH-R and its splice variants, on different types of cancer cell lines has been demonstrated [37–39]. In our study, we found association neither between GHRH-R expression and the HPV status, nor between GHRH-R expression and response to treatment.

## Conclusion

In conclusion, the present study found that one-fifth of the patients with ESCC proved to have HPV-positive tumors in Hungary’s Southwestern region. HPV positivity was accompanied by significantly increased expressions of Hsp 90 and 16.2. HPV-positive cases and cases expressing high intensity Hsp 90 and 16.2 levels showed a significantly poorer response to oncological treatment and worse overall survival. We admit the limitations of our study. Given the limited sample size, the results of this report should be interpreted with caution. To confirm the significance of our observation further larger scale studies are needed.

## Abbreviations

ACIP: Advisory Committee on Immunization Practices
CISH: Chromogenic in situ hybridization
CRT: Chemoradiotherapy
CT: Computer tomography
ESCC: Esophageal Squamous Cell Carcinoma
GH: Growth hormone
GHRH: Growth hormone-releasing hormone
GHRH-R: Growth hormone-releasing hormone receptor
HPV: Human papillomavirus
Hsp: Heat shock protein
IGF-I: Insuline-like growth factor I
RECIST: Response evaluation criteria in solid tumors
